# Dipyridamole-loaded 3D-printed bioceramic scaffolds stimulate pediatric bone regeneration *in vivo* without disruption of craniofacial growth through facial maturity

**DOI:** 10.1038/s41598-019-54726-6

**Published:** 2019-12-05

**Authors:** Maxime M. Wang, Roberto L. Flores, Lukasz Witek, Andrea Torroni, Amel Ibrahim, Zhong Wang, Hannah A. Liss, Bruce N. Cronstein, Christopher D. Lopez, Samantha G. Maliha, Paulo G. Coelho

**Affiliations:** 1 Department of Biomaterials & Biomimetics, NYU College of Dentistry, 433 1st Avenue, New York, NY 10010 USA; 2 Hansjörg Wyss Department of Plastic Surgery, NYU Langone Health, 307 E 33rd St, New York, NY 10016 USA; 3 Department of Medicine, NYU Langone Health, 550 1st Avenue, New York, NY 10016 USA; 4 Department of Mechanical Engineering, NYU Tandon School of Engineering, 6 MetroTech Center, Brooklyn, NY 11201 USA; 5 Department of Plastic and Reconstructive Surgery, Johns Hopkins School of Medicine, 601 N Caroline St, Baltimore, MD 21205 USA; 6 Department of Plastic Surgery, University of Pittsburgh Medical Center, 3601 Fifth Ave, Pittsburgh, PA 15213 USA

**Keywords:** Implants, Biomedical materials, Regenerative medicine, Tissue engineering, Translational research

## Abstract

This study investigates a comprehensive model of bone regeneration capacity of dypiridamole-loaded 3D-printed bioceramic (DIPY-3DPBC) scaffolds composed of 100% beta-tricalcium phosphate (β –TCP) in an immature rabbit model through the time of facial maturity. The efficacy of this construct was compared to autologous bone graft, the clinical standard of care in pediatric craniofacial reconstruction, with attention paid to volume of regenerated bone by 3D reconstruction, histologic and mechanical properties of regenerated bone, and long-term safety regarding potential craniofacial growth restriction. Additionally, long-term degradation of scaffold constructs was evaluated. At 24 weeks *in vivo*, DIPY-3DPBC scaffolds demonstrated volumetrically significant osteogenic regeneration of calvarial and alveolar defects comparable to autogenous bone graft with favorable biodegradation of the bioactive ceramic component *in vivo*. Characterization of regenerated bone reveals osteogenesis of organized, vascularized bone with histologic and mechanical characteristics comparable to native bone. Radiographic and histologic analyses were consistent with patent craniofacial sutures. Lastly, through application of 3D morphometric facial surface analysis, our results support that DIPY-3DPBC scaffolds do not cause premature closure of sutures and preserve normal craniofacial growth. Based on this novel evaluation model, this DIPY-3DPBC scaffold strategy is a promising candidate as a safe, efficacious pediatric bone tissue engineering strategy.

## Introduction

## Pediatric craniofacial reconstruction

Defects of the pediatric craniofacial skeleton can occur due to congenital, neoplastic, infective, traumatic, or iatrogenic causes^1^. Cleft palate and craniosynostosis, or premature fusion of the cranial sutures, for instance, are two of the most common congenital causes of craniofacial bone defects/deformities and account for 1 in 600 and 1 in 2000 live births respectively. Affected patients commonly require bony reconstruction at the time of active facial growth and surgical interventions are designed to restore the craniofacial skeleton while limiting impairment to face and skull development. Furthermore, these deformities and required surgical interventions are a source of psychosocial and financial burden not only for patients but families and healthcare systems due to their prevalence and treatment complexity associated with this population^1–4^.

Autologous bone grafting remains the standard of care for reconstruction of the pediatric craniofacial skeleton^1,5–7^. Key limitations of autologous bone graft include donor site morbidity, bone resorption, limited quantity and shape of bone stock, as well as difficulty in shaping bone grafts into complex 3-dimensional forms such as those often needed to reconstruct craniofacial defects^5,7–11^. While alternatives to autologous tissues such as allograft or foreign-body implants can ameliorate some of these challenges, they pose their own restrictions including inflammation, infection, extrusion, fragmentations and inability to grow or entirely integrate with the child’s growing skeleton^1,6,7^.

## Pediatric bone tissue engineering

Due to the limitations of current reconstructive options, there exists growing interest in bone tissue engineering and regeneration strategies for pediatric craniofacial reconstruction, including the application of bioactive scaffolds, osteogenic pharmacologic agents, and stem cell therapies. Despite relative advances in adult craniofacial bone tissue engineering^12–15^, an optimal approach has yet to be demonstrated for use in the pediatric population^7,16,17^. Compared with adult patients, the thickness of pediatric bone is thinner^18^, and there is reduction in the osteoinductive potential of the dura after 12 months^9,19^ which impairs native regeneration of bony defects. Moreover, long-term growth and development of the craniofacial skeleton must be carefully considered since even with gold-standard treatment, patients may experience growth restriction and require multiple revision surgeries^17^. An ideal pediatric bone replacement or regenerative therapeutic agent would be biocompatible, patient specific, bioresorbable, lead to the regeneration of mature, vascularized bone and ultimately restore form and function of the skeleton without impeding facial development^16,18^. Due to these unique considerations, efficacious translation of bone tissue engineering strategies in a pediatric context have been limited and investigation into pediatric skeletal tissue engineering strategies remains in its infancy^20,21^. We seek to evaluate a novel tissue regeneration strategy that may begin to fulfill these criteria for successful translation to a pediatric craniofacial context by investigating both a cleft and calvarial surgical defect model.

## Dipyridamole 3D-printed bioceramic scaffolds

Our approach combines customizable 3D-printed bioceramic (3DPBC) scaffolds and dipyridamole, a novel osteoinductive agent. By utilizing computer aided design/manufacturing (CAD/CAM), scaffolds can be precisely designed and manufactured to the specifications of the patient’s craniofacial defect^12,22^. The scaffolds described in this study comprised of 100% beta-tricalcium phosphate (β -TCP) to provide structure and rigidity to the defect, promote osteocyte migration and osteoconduction within scaffold interstices, and demonstrate better biodegradability compared to other commonly used biomaterials such as hydroxyappetite (HA)^12^, thus leaving uninterrupted regenerated bone.

Dipyridamole (DIPY), an indirect adenosine 2A receptor (A2AR) agonist, has been utilized to augment the osteoinductive potential of these bioceramic scaffolds. DIPY blocks cellular adenosine uptake by the purine transporter ENT1, thereby increasing extracellular adenosine. A2AR activation has been shown to stimulate osteoblast proliferation and differentiation *in vitro*^23^ and *in vivo*^24^. Furthermore, dipyridamole has a long history of safe use in pediatric patients, with decades of safe systemic administration exceeding the systemic exposure resulting from local delivery for osteogenesis^23,25–28^.

Our group has previously demonstrated that 3DPBC scaffolds significantly generate vascularized bone *in vivo* across critically-sized defects compared to unfilled controls, which do not regenerate both in adult and pediatric translational animal models^22,27–31^. Dipyridamole has been shown to significantly augment the osteogenic capacity of 3DPBC scaffolds compared to scaffolds without dipyridamole^24,32,33^. Importantly, optimization studies have identified a dose of 1,000 µM to maximally promote osteogenic regeneration when used in combination with 3DPBC scaffolds in animal models without observed negative side-effects including premature suture fusion or exuberant bone formation in the short-term^27,28^.

While these pilot studies have demonstrated safety of this approach in the immature craniofacial skeleton, they have been limited to short term analyses of scaffolds compared to unfilled defects^27,28^. A foremost concern in clinical pediatric craniofacial reconstruction is the effects of interventions on development of the facial skeleton at craniofacial maturity. Longitudinal demonstration of scaffold osteogenesis, favorable degradation over time, and safety from immaturity to skeletal maturity are necessary for successful translation to and adoption in a pediatric craniofacial context. Finally, while scaffold superiority over null interventions has been demonstrated in previous reports^27,29,31^, direct comparison of scaffold osteogenesis compared to standard of care interventions such as autogenous bone graft has yet to be performed.

This study employs two different models of pediatric craniofacial defects and reports a 6-month analysis of the effect of DIPY-3DPBC scaffolds on osteogenic regeneration compared to autogenous bone graft and measures the effects of this bone tissue engineering construct on the development of the growing craniofacial skeleton throughout and beyond the period of skeletal maturity.

## Results

### DIPY-3DPBC Scaffolds promote volumetrically significant osteogenic regeneration of calvarial and alveolar defects. Regeneration is comparable to or greater than standard of care treatment with favorable degradation of bioactive ceramic component *in vivo*

**Figure 1:**
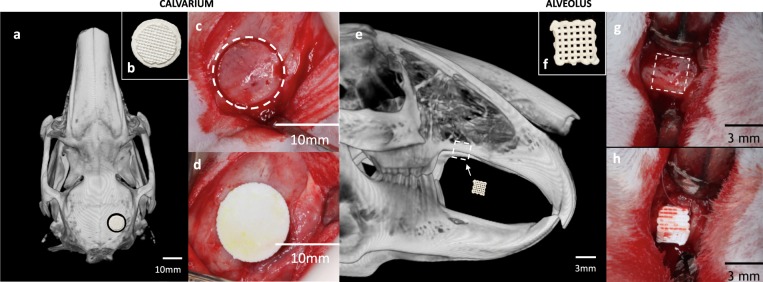
**(a)** Schematic location of unilateral calvarial defect. (**b)** Inferior view of printed calvarial scaffold **(c)** intraoperative location of calvarial trephine **(d)** fit-and-fill reconstruction of calvarial defect with scaffold. **(e)** Schematic location of unilateral alveolar defect (**f)** lateral view of printed calvarial scaffold **(g)** intraoperative location of alveolar defect **(h)** fit-and-fill reconstruction of alveolar defect with scaffold.

**Figure 2:**
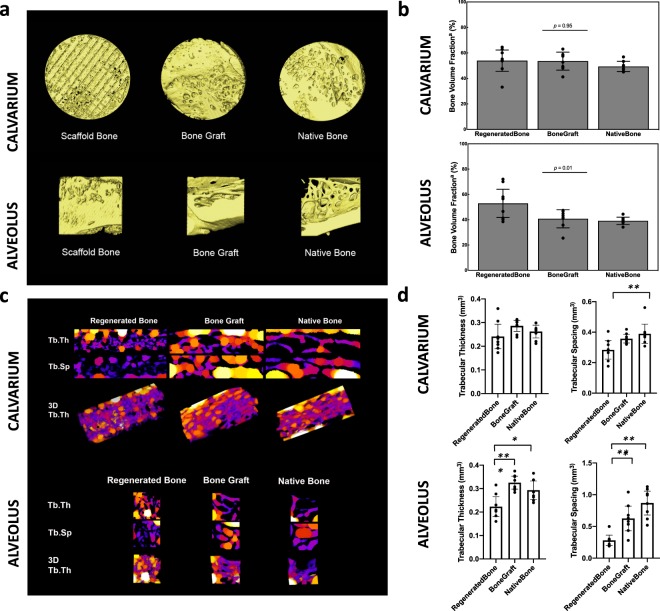
3D reconstructions and volumetric analysis of regenerated bone at 24 weeks *in vivo*. **(a)** 3D reconstruction of scaffold-regenerated bone (yellow) demonstrates regeneration across entire span of trephined defect and demonstrates comparable morphology compared to bone graft and un-operated native bone. **(b)** Volumetric analysis shows comparable bone volume fraction regenerated by scaffold in the calvarium compared to bone graft and native bone, and greater bone volume fraction regenerated by scaffold in the aveolus compared to bone graft and native bone. (**c)** 2D and 3D heat maps of Trabecular Thickness (Tb.Th) and Trabecular Spacing (Tb.Sp) of Regenerated Bone, Bone Graft, and Native Bone in the calvarium and alveolus. Brighter colors represent greater Tb.Th or Tb.Sp. (**d)** Quantification of Tb.Th and Tb.Sp across samples for the calvarium and alveolus. ^a^Bone Volume Fraction = (Bone Volume/Total Volume). Error bars are 95% confidence intervals. *p < 0.05, **p < 0.01, ***p < 0.001.

**Figure 3:**
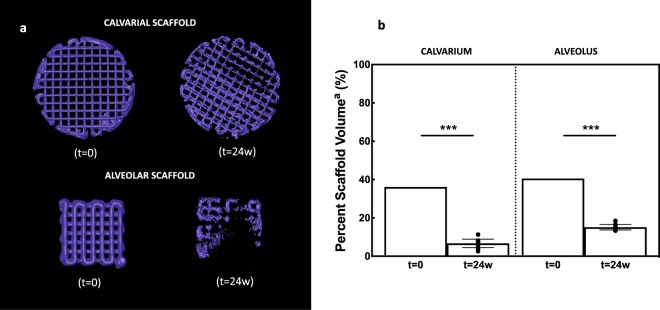
3D reconstructions and volumetric analysis of scaffold at 24 weeks *in vivo* **(a)** 3D reconstruction and **(b)** percent scaffold volume of scaffold component in the cleft and alveolus show significant degradation of the scaffold (purple) component between t = 0 and t = 24 weeks. ^a^Percent Scaffold Volume = (Scaffold Volume/Total Volume). Error bars are 95% confidence intervals. ***p < 0.001.

Amira software reconstruction was used to quantify and visualize new bone formation and scaffold occupancy of calvarial and alveolar defects repaired with DIPY-3DPBC scaffolds (Fig. 1). After 24 weeks, calvarial defects repaired with DIPY-3DPBC scaffolds regenerated an average bone volume fraction (bone volume/total volume) of 53.9 ± 8.4% (Fig. 2b). This was significantly increased compared to previously reported bone volume fraction regenerated by scaffolds at 8 weeks^28^ (30.3 ± 14.3, p = 0.005) and comparable to volume of bone volume fraction of autogenous bone graft and un-operated calvarium (53.5 ± 7.6% and 49.4 ± 3.9%; p = 0.95; Fig. 2b). Analysis of degradation kinetics revealed calvarial scaffold volume fraction (scaffold volume/total volume) of 15.1 ± 1.5% at 24 weeks. This was a significant decrease from originally implanted scaffold volume fraction of 36.1% (*p* < 0.001; Fig. 3).

In the alveolus, defects repaired with DIPY-3DPBC scaffolds also regenerated bone volumes significantly increased compared to previously reported bone volumes of scaffolds at 8 weeks^33^ (20.0 ± 8.2, p = 0.001) this was significantly increased compared to bone graft and un-operated bone (52.9 ± 7.1% vs. 40.7 ± 8.5% vs. 39.3 ± 4.1%; ANOVA *p* = 0.01 Fig. 2b). There was also significant degradation of the scaffold component in the alveolus at 24 weeks compared to originally implanted scaffold volume fraction (6.7 ± 3.5% vs. 40.6%; *p* < 0.001; Fig. 3).

In the calvarium, trabecular thickness was not different between regenerated bone, bone graft, or native bone. Trabecular spacing was lower in regenerated bone compared to native bone (p = 0.008). In the alveolus, trabecular thickness was lower in regenerated bone compared to bone graft (p < 0.001) and native bone (p = 0.01). Trabecular spacing was also lower in regenerated bone compared to bone graft (p = 0.006) and native bone (p < 0.001). These findings may represent bone regeneration filling the dimensions of the implanted scaffold and increased anabolic activity in response to the higher-load conditions of the alveolus compared to the calvarium^34^. We suspect that bone undergoing subsequent remodeling may demonstrate more physiologic trabecular morphology in longer-term studies as rabbits age and scaffold contribution to biomechanics decrease^35,36^.

### DIPY-3DPBC Scaffolds promote osteogenesis of organized, vascularized bone with histologic and mechanical characteristics comparable to native bone

**Figure 4:**
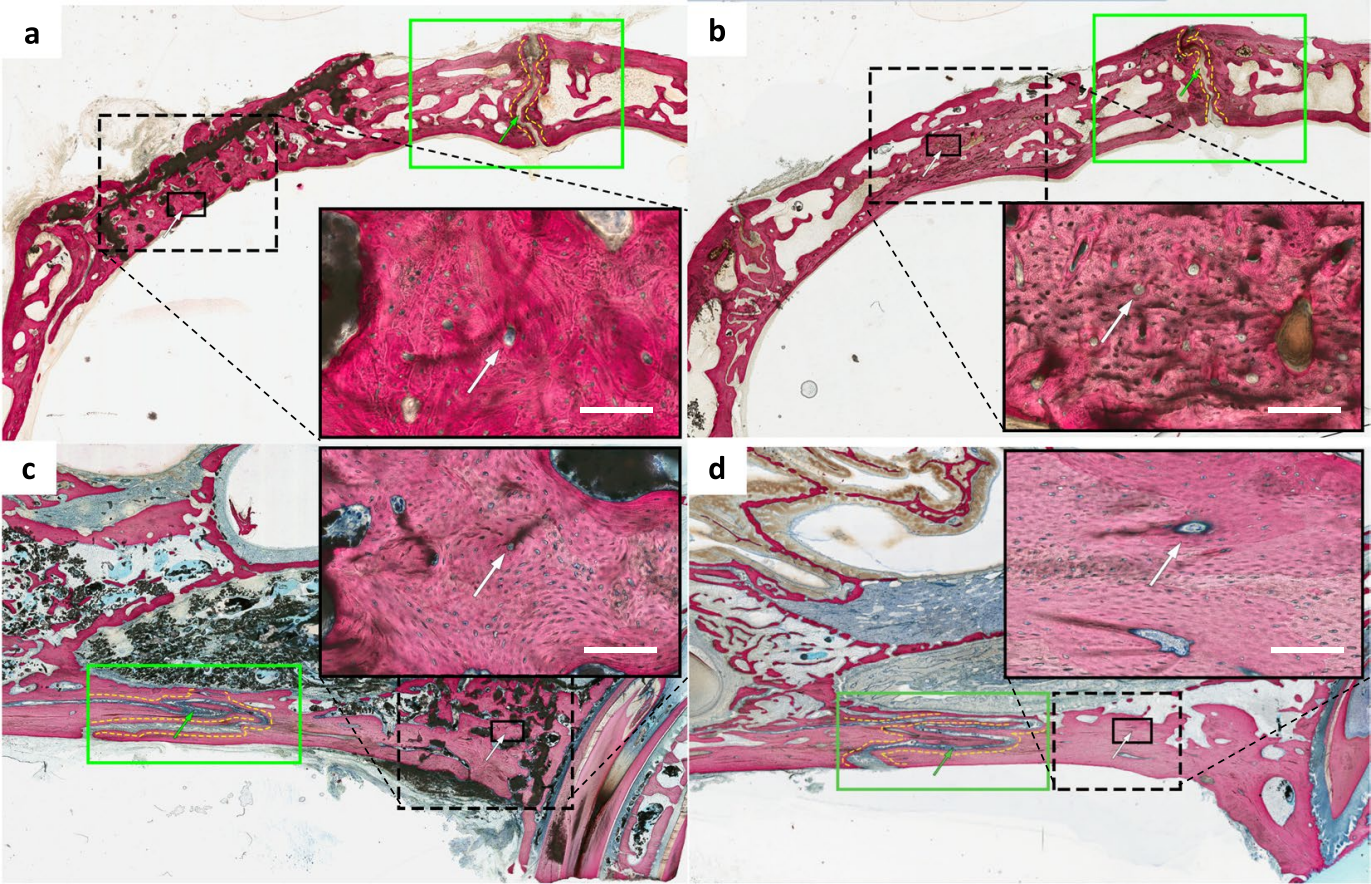
Non-decalcified histology of **(a)** calvarial and **(c)** alveolar scaffolds and (**b)** calvarial and **(d)** alveolar bone graft showed blood vessels stained with stevenel’s blue with surrounding organized bone (white arrows). Patent sutures without evidence of disruption or premature fusion (green arrows) were visualized adjacent to calvarial and alveolar reconstruction in all samples without evidence of ectopic bone formation or inflammatory cells. All scale bars = 1 mm.

**Figure 5:**
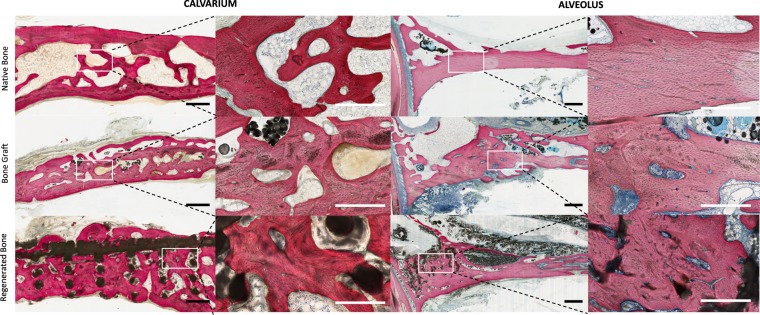
Characterization of bone morphology in the calvarium (left) and alveolus (right). Scaffold-regenerated bone (top) in both defect areas result in histologically-organized bone that evidences many features of native bone (bottom) comparable to regeneration by bone graft (middle). All scale bars = 1 mm.

Non-decalcified histologic sections of defects stained with Van Giesen’s red revealed bone spanning the entirety of calvarial and alveolar defects (Fig. 4). Bone was observed between scaffold interstices and was contiguous with unoperated surrounding calvarial and alveolar bone (Fig. 4). Throughout samples, all cranial and maxillary sutures were patent (Fig. 4). There was no evidence of ectopic bone, excess inflammatory cells, or scaffold fragmentation (Figs. 4 and 5).

Characterization of bone from corresponding un-operated, contralateral calvarium and alveolus revealed differences in cellular organization of bone. In the calvarium, bone demonstrated primarily trabecular organization characteristic of cancellous bone. The periosteal and dural aspects of the calvarial shelf demonstrated lamellar orientation parallel to external calvarial curvature. Between these plates, spaces with adipose tissue characteristic of diploic bone marrow cavities were observed (Fig. 5). In the alveolus, bone along the inferior aspect of the ridge demonstrated organization with evidence of haversian canals characteristic of compact bone. Superiorly, and directly anterior to the premolar tooth root, alveolar bone appeared trabecular, with marrow-spaces consistent with cancellous bone.

Defects repaired with DIPY-3DPBC scaffolds revealed both lamellar organization and formation of trabeculae consistent with cancellous bone maturation as well as maturation of compact bone, with formation of osteons with Haversian canals containing micro vessels stained with Stevene’s blue (Fig. 5). In the calvarium, bone regenerated by DIPY-3DPBC scaffolds resulted in more consistent lamellar organization and formation of bone spicules with adipose-filled diploic spaces which more closely resembles native calvarium compared to bone-graft regenerated bone, which appeared to form more dense regions of compact bone with compact osteonic rather than trabecular organization (Fig. 5). In the alveolus, DIPY-3DPBC scaffolds resulted in bone that was morphologically similar to bone-graft regenerated bone and native bone, though both bone graft and scaffold-reconstructions demonstrated a higher degree of neovascularization and evidence of cellular remodeling compared to native bone (Fig. 5).

**Figure 6:**
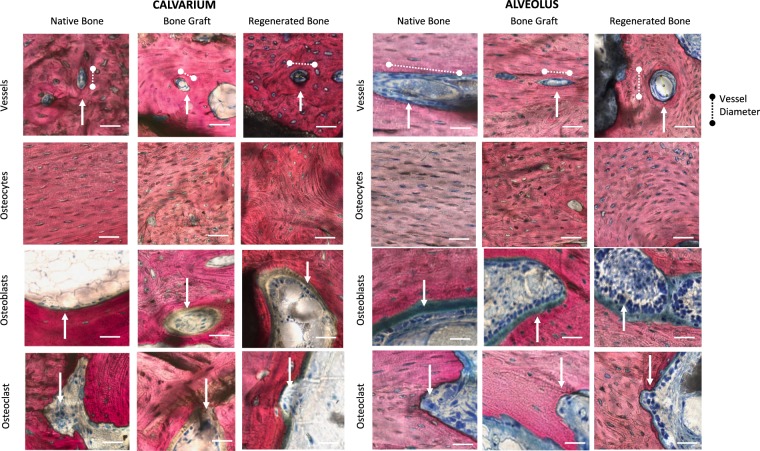
Representative images of vessel density, vessel diameter, osteocyte denstity, osteoblast, and osteoclast density were quantified to demonstrate bone healing and neovascularization of regenerated bone across groups. **(b)** Quantification of histologic organization and maturity of scaffold-regenerated bones versus bone graft and native bone. No statistically significant difference between scaffold-regenerated bone and bone graft or native bone in any of these features. CI = confidence Intervals. All scale bars = 100 µm.

Across samples, bone in all conditioned demonstrated characteristics of mature, organized vascular and cellular components of bone (Fig. 6). Histologic quantification did not reveal any significant differences in vessel density for bone regenerated by DIPY-3DPBC scaffolds compared to bone graft reconstruction and un-operated, native bone (Table 1). In the alveolus, vessel density was also statistically homogeneous between all groups. One way ANOVA and multiple comparisons of means revealed no statistical differences in average vessel diameter, though in the alveolus, scaffold-regenerated bone trended towards smaller vessels compared to bone graft and unoperated native bone, suggesting increased degree of neovascularization in this condition.

**Table 1:** Quantification of histologic organization and maturity of scaffold-regenerated bones versus bone graft and native bone.

	Native Bone (95% CI)	Bone Graft (95% CI)	Scaffold Bone (95% CI)	p-value^a^
**CALVARIUM**				
Vessel Density (/mm^2^)	**0.27** (0.13–0.42)	**0.27** (0.07–0.47)	**0.19** (0.03–0.35)	**0.54**
Vessel Diameter (µm)	**36.9** (3.0–70.8)	**60.8** (31.7–89.8)	**52.2** (37.2–67.2)	**0.16**
Osteocyte Density (/mm^2^)	**157.3** (129.1–185.6)	**190.4** (143.9–237.0)	**157** (104.5–209.6)	**0.20**
Osteoblast Density (/mm^2^)	**0.65** (−0.89–2.20)	**2.61** (−3.16–8.38)	**3.92** (−1.20–9.04)	**0.29**
Osteoclast Density (/mm^2^)	**0.01** (−0.039–0.063)	**0.06** (−0.023–0.15)	**0.06** (−0.017–0.13)	**0.41**
Osteoblast/Osteoclast (ratio)	**54.69**	**41.71**	**68.60**	**0.44**
**ALVEOLUS**				
Vessel Density (/mm^2^)	**0.57** (0.22–0.93)	**0.63** (0.27–0.99)	**0.71** (0.29–1.14)	**0.72**
Vessel Diameter (µm)	**108.8** (87.9–129.8)	**82.6** (54.5–110.7)	**76.0** (12.4–139.6)	**0.20**
Osteocyte Density (/mm^2^)	**148.1** (141.9–154.3)	**166.5** (115.6–217.4)	**153.6** (121.6–185.6)	**0.50**
Osteoblast Density (/mm^2^)	**5.84** (−1.60–13.29)	**6.76** (−8.74–22.27)	**16.95** (−2.42–36.31)	**0.18**
Osteoclast Density (/mm^2^)	**0.11** (−0.080–0.31)	**0.06** (−0.043–0.16)	**0.26** (−0.017–0.53)	**0.21**
Osteoblast/Osteoclast (ratio)	**51.12**	**113.61**	**65.47**	**0.25**

No statistically significant difference between scaffold-regenerated bone and bone graft or native bone in any of these features. CI = confidence Intervals. ^**a**^p-value by one-way ANOVA.

Osteocyte density analysis revealed mean calvarial scaffold osteocyte was statistically equivalent across groups (*p* = 0.20; Fig. 5b). Alveolar mean osteocyte count was likewise equivalent across groups (*p* = 0.50; Table 1). In all conditions, osteoblast density was many folds greater than osteoclast density (Table 1). This is consistent with intact, mature healing bone not undergoing significant resorption.

**Figure 7:**
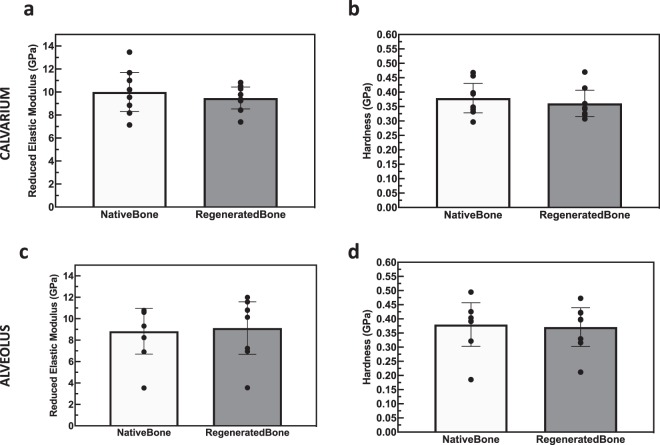
Reduced Elastic Modulus (Er) of **(a)** calvarial and **(c)** alveolar scaffold-regenerated bone shows no difference compared native control. Hardness (H) of **(b)** calvarial and **(d)** alveolar scaffold-regenerated bone shows no difference compared native control. Error bars are 95% confidence intervals.

Assessment in the calvarium, of the reduced elastic modulus (Er) and hardness (H), acquired by means of nanoindentation displayed an Er of 9.5 ± 1.7 GPa and H of 0.36 ± 0.05 for scaffold-regenerated bone, which yielded to be statistically homogenous with native bone analyzed outside of scaffold interstices (Er: 10.01 ± 1.7, *p = *0.53; Fig. 5b: H: 0.36 ± 0.05, *p* = 0.54; Fig. 7a,b). Likewise, analysis of the native and regenerated bone of the alveolus revealed statistically similar Er and H of scaffold-regenerated bone and native bone (Er: 9.1 ± 2.5 GPa vs. 8.8 ± 2.1 GPa, *p* = 0.83; H: 0.37 ± 0.07 GPa vs. 0.38 ± 0.08 GPa, *p* = 0.84; Fig. 7c,b).

### DIPY-3DPBC scaffolds do not cause premature closure of craniofacial sutures and do not result in disruption of facial symmetry

**Figure 8:**
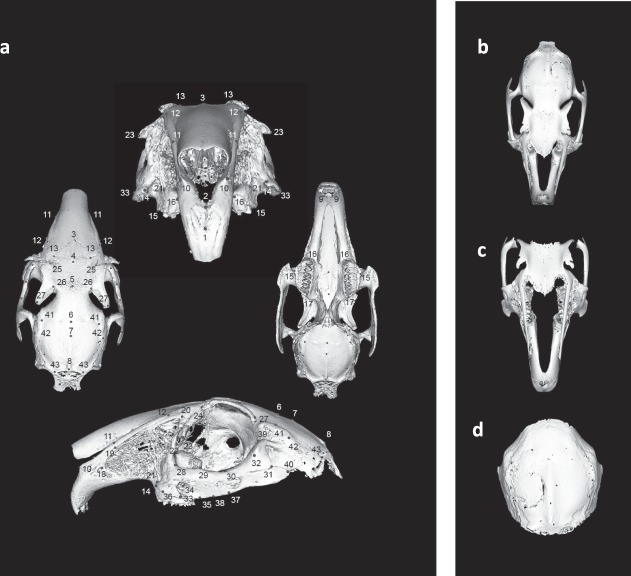
**(a)** Example of model landmarking for rabbit craniofacial surface for model building. Basemesh models for **(b)** global facial model, **(c)** isolated midface model, and **(d)** isolated calvarial models of facial symmetry for rabbits af the age of skeletal maturity.

Detriment to facial development secondary to implantation of DIPY-3DPBC scaffolds to the right side of the craniofacial skeleton may manifest as asymmetric growth. The higher asymmetry indices should represent greater degree of asymmetry which could result from premature fusion of craniofacial sutures. Using 3D morphometric analysis (Fig. 8), we did not detect any significant differences in global asymmetry index for animals treated with scaffold, bone graft animals and un-operated controls (Table 2). Multiple comparisons with Tukey’s correction indicated that scaffold-treated animals did not present an increased asymmetry in comparison to those treated with autogenous bone graft or controls.

**Table 2:** Morphometric analysis for global facial model, isolated midface model, and isolated calvarial models of facial symmetry for rabbits af the age of skeletal maturity.

	AI	95% LCI	95% UCI	*p-*value
**Global Asymmetry Model**				
Control	132.2 ± 23.2	109.0	155.5	0.1448^a^
Bone Graft	140.3 ± 7.1	133.2	147.4	
Scaffold	122.1 ± 19.0	103.1	141.2	
**Isolated Midface Model**				
Control	133.5 ± 22.1	111.4	155.6	0.3467^a^
Bone Graft	137.9 ± 10.8	127.1	148.6	
Scaffold	124.7 ± 18.0	106.7	142.8	
**Isolated Calvarial Model**				
Control	54.2 ± 33.3	20.97	87.51	0.381^a^
Bone Graft	68.8 ± 23.1	45.77	91.87	
Scaffold	52.2 ± 17.1	35.08	69.24	

Morphometric analysis demonstrates no significant difference between means in Asymmetry Index (AI) between conditions. **LCI:** Lower bound of Confidence Interval, **UCI:** Upper bound of Confidence Interval. ^**a**^p-value by one-way ANOVA.

In construction of an isolated midface model seeking to target local asymmetry resulting from the disruption of the maxillary suture, there were no significant differences between animals treated with scaffolds, bone graft, or un-operated controls (Table 2). Likewise, in an isolated calvarial model evaluating local asymmetry resulting from disruption of the calvarial sutures, there was no significant difference in asymmetry index between scaffold-treated animals, bone graft-treated animals, or un-operated controls (Table 2).

3D morphometric analysis was validated using 2-dimensional cephalometric measurements. Comparative cephalometric measurements of rations of operated and un-operated sides yielded no significant differences in symmetry between scaffold-repaired animals, bone graft animals or un-operated controls (Table 3).

**Table 3:** Cephalometric ratios of operated and unoperated sides show no difference in symmetry between conditions.

	Control	95% LCI	95% UCI	Bone Graft	95% LCI	95% UCI	Scaffold	95% LCI	95% ULCI	p-value
**Cephalometric Measurement Asymmetry Index**										
**ML**	**0.985 ± 0.007**	0.978	0.992	**0.991 ± 0.020**	0.971	1.011	**0.996 ± 0.019**	0.977	1.014	0.66^a^
**MH**	**0.975 ± 0.034**	0.941	1.010	**0.986 ± 0.028**	0.958	1.013	**1.000 ± 0.039**	0.961	1.039	0.52^a^
**CL**	**0.999 ± 0.082**	0.917	1.081	**0.972 ± 0.045**	0.927	1.017	**1.051 ± 0.070**	0.981	1.122	0.10^a^
**CW**	**0.996 ± 0.064**	0.932	1.060	**0.990 ± 0.084**	0.906	1.073	**1.002 ± 0.070**	0.932	1.071	0.96^a^

**ML**: Maxillary Length (anterior maxillary premolar – anterior corpus ossis incicivi); **MH**: Maxillary Height (anterior corpus ossis incicivi – posterior alveolus I3); **CL**: Calvarial Length (temporozygomatic intercept — occipital intercept); **CW**: Calvarial width (bregma — temporozygomatic intercept); **LCI:** Lower bound of Confidence Interval, **UCI:** Upper bound of Confidence Interval; ^a^p-value by one-way ANOVA.

## Discussion

Challenges specific to pediatric craniofacial skeletal reconstruction, particularly limitations to current standard of care interventions (autologous bone graft) result in opportunities and inspire investigation of alternative strategies for bone tissue engineering. In patients affected by cleft lip and palate, reconstruction of an alveolar (maxillary) cleft can ensure proper tooth eruption, maxillary arch stability, and appropriate midface development. Unfortunately, autogenous bone graft in reconstruction of both the alveolus and calvarium is limited by survival, donor site morbidity and added healthcare cost associated with extended operative time and increased length of stay for pain control associated with bone graft harvest^7^. Although alloplastic materials are a potential alternative, these implants do not grow with the child, increasing the likelihood of need for replacement at a later age. Both pediatric calvarial and maxillary reconstruction lend themselves to augmentation of innate healing processes given limitations with currently used modalities. However, currently employed bone tissue engineering strategies such as bone matrix substitute, synthetic polymer scaffolds, and morphogenic proteins are still limited by subsequent graft resorption, inability to fill critically sized defects, and reported disruption of suture growth^6,9,20,27,37,38^.

We propose that DIPY-3DPBC scaffolds offer several advantages to clinical standard of care and published bone tissue engineering strategies alike: 1) custom design via CAD/CAM of β –TCP allows for personalized reconstruction of large defects compared to bone graft or porous biomaterials and also specification of meso and nanostructure to optimize bone regeneration, 2) composition of 100% β –TCP allows for improved degradation of inorganic scaffold component compared to hydroxyapatite (HA) scaffolds^12^, which may obviate the need for reoperation in a pediatric population, and 3) use of dipyridamole is safe and preserves normal craniofacial growth in this high-risk population.

We present a novel, multimodal investigation including radiographic, histologic, biomechanical, and facial symmetry analyses to directly develop and assess these clinically-translatable bone-engineering advantages for pediatric patients. To our knowledge, the current study is the longest *in vivo* investigation of DIPY-3DPBC scaffolds to date, and our results bring to bear the above advantages of dipyridamole 3DPBC scaffolds. Our previously published investigations demonstrate that DIPY-3DPBC scaffolds regenerate defects that are critically-sized, or otherwise have been demonstrated to prevent spontaneous bony healing^27^. Here, we add to prior studies to demonstrate that dipyridamole-coated 3DPBC tissue engineering construct can generate vascularized bone with comparable volumetric density as autogenous bone graft, the clinical standard of care, as well as native bone. We used radiographic assessment as our primary endpoint, as this would be the clinical means of evaluating this technology, and with it we were able to determine that scaffold degradation kinetics demonstrated progressive absorption of the scaffold and replacement with regenerated bone. We then applied human bone tissue engineering assessments with regards to vascular, cellular, and biomechanical criteria for mature, successful bone regeneration, which further underscored viability of this approach compared to native bone and gold-standard treatment after 24 weeks *in vivo*^39,40^.

Most importantly, no adverse effects on cranial sutures or craniofacial growth were noted in the study animals, which developed through the time of craniofacial skeletal maturity. Physiologic and clinical relevance was of critical importance in the application this model to an often difficult to assess population. Facial maturity in our model was defined conservatively at 28–30 weeks of age^41^. Morphometric analysis has previously been used to describe patterns of growth, shape and development in unaffected and dysmorphic faces in syndromic human pediatric populations^42,43^. Application of this to animal study provided a means to concurrently analyze size and form of developing rabbit skulls. Previous animal models of experimentally-induced unilateral craniofacial suture restriction results in facial asymmetry between affected an un-affected sides^44^. Further, investigation of unilateral coronal synostosis and anterior synostotic plagiocephaly in humans suggests that premature closure of cranial and midface sutures can result in asymmetry of the calvarium as well as the midface^45–47^. In our model, even with close proximity of the surgical defects to developing sutures, DIPY-3DPBC scaffolds do not produce an increased craniofacial asymmetry compared to those reconstructed with bone graft or un-operated controls. It is notable that previous studies utilizing the same growing rabbit model demonstrated premature fusion of the cranial suture and restricted cranial growth when cranial defects were treated with BMP-2^48^. BMP-2 is a commonly used osteogenic agent in pediatric craniofacial reconstruction despite growing evidence that its use can result in premature suture fusion and facial growth disruption^48–50^. As noted previously, growth restriction is a serious complication of pediatric craniofacial reconstruction that challenges autogenous reconstruction, autogenous implants, and bioceramic tissue engineering alike^9,27,38^. The lack of facial asymmetry demonstrated in this sample, suggests that DIPY-3DPBC scaffolds do not negatively affect facial suture patency or craniofacial development. Our 3D morphometric analysis is consistent with 2D reference measurements used clinically in evaluation of the maxilla and calvarium, supporting the novel application of this 3D modeling strategy for assessment of facial symmetry in a translational animal model.

Our surgical injury protocol was applied to two distinct surgical sites, to model the various complex clinical applications and to acknowledge the functionally different considerations of calvarial and alveolar reconstruction. We selected alveolar and calvarial reconstruction as they represent a significant clinical burden for pediatric craniofacial patients as well as areas of active tissue engineering research. Craniofacial growth and development is affected by complex genomic, metabolic, and mechanical factors — often patients require multiple craniofacial procedures before skeletal maturity. We believe that the success of our model at both defect sites supports the efficacy and clinical translatability of this technology.

For example, this study employed a simplified model of bone graft in approximating the complexity and size of calvarial defects treated in the clinical arena. Cranial vault remodeling often requires significant resection and reconstruction in irregular geometries that may rely on alloplastic reconstruction given limited stock and shape of donor bone. Further, skull thickness and regenerative capacity may vary based on anatomic location of cranial reconstruction. It is unlikely that in a clinical scenario, an autograft with the precise dimensions and curvature of the reconstructed defect would be used in the fashion described in this report. As such, the calvarial bone graft results achieved in our investigation may represent idealized outcomes of results for cranial reconstruction. As a result, the equivalence of DIPY-3DPBC scaffold reconstruction to this idealized bone graft model in our study underscores the potential advantages of DIPY-3DPBC scaffolds, as an inherent benefit of the technology is the ability to design and implement custom-fitted implants tailored to complex reconstructive challenges^12^.

One limitation of our model is that we do not directly investigate the repair of congenital facial clefts —instead, we employ a surgically created cleft model in order to approximate the treatment of congenital clefts commonly presenting in pediatric patients. Our aim was to investigate bony regeneration of surgical defects in this pediatric model, which we believe remains relevant in the treatment of congenital cleft patients, as the opposing alveolar ridge may be trimmed in preparation for filling by bone graft in congenital cleft patients^51,52^. Given our demonstration of successful regeneration in this model, the investigation of bony regeneration in a congenital cleft animal model and finally the study of regeneration in syndromic clefts remain important further areas of investigation.

Future studies will also be aimed at long-term analysis of bone quality following the completion of scaffold resorption in order to accurately estimate scaffold degradation kinetics and the continued viability and morphology of regenerated bone. Also, we acknowledge that rodent models typically represent idealized models of bone regeneration technologies due to favorable bone metabolism and scale, this is a limitation inherent to translational research, especially given important concerns about safety in pediatric clinical trials. As such, we also intend to investigate the use of these biomaterials in reconstructing larger defects in a swine animal model as well human *in vitro* 3D culture models.

Ultimately, we uniquely demonstrate that DIPY-3DPBC scaffolds generate vascularized bone with radiographic, histologic, and mechanical properties similar to native bone as defined by clinically-relevant outcomes — all without impediment to normal craniofacial skeletal development. Thus, we believe the DIPY-3DPBC scaffolds presented in this report may overcome limitations of autologous bone graft and begin to fulfill context-specific requirements of an effective pediatric bone tissue engineering strategy.

## Materials and Methods

### Subjects, surgical injury model, and reconstruction

All experiments were approved by the NYU School of Medicine Institutional Animal Care and Use Committee (IACUC). Experiments were performed in accordance with the NYU School of Medicine Institutional Animal Care and Use Committee (IACUC) standards. Sixteen (n = 8 per group) four-week-old (skeletally immature) New Zealand White rabbits underwent surgical creation of two unilateral right-sided defects: in the calvarium and alveolus (see supplementary information for study groups). Defects were repaired with either custom DIPY-3DPBC scaffolds or autogenous bone graft.

In the calvarium, 10 mm-diamter defects were created^28^. First, a 13 mm skin incision was made on posterior scalp to the right of midline in order to visualize the rabbit’s calvarium. Soft tissue and periosteum were dissected to expose calvarial bone. Defects were then created uniformly, located 2 mm posterior and lateral to the coronal and sagittal sutures, respectively, using at 10 mm diameter trephine (Fig. 1b). Using a fit-and-fill method, defects were repaired with either 3D-printed scaffolds loaded with 1,000 µM DIPY or bone graft. Proper inset was ensured by obtaining primary stability of the scaffold and avoiding violation of the dura mater.

Calvarial bone graft was created by immersing trephined calvarium in saline and returning the bone graft to the defect. Once inset was confirmed, the soft tissue envelope was closed in layered fashion.

In the alveolus, a 13 mm skin incision was created on the right aspect of the midface. Soft tissue and periosteum were dissected to visualize the maxilla, the alveolar ridge and the maxillary suture. Using 3D printed template, uniform 3.5 mm × 3.5 mm defects were created using oral surgery burr 2 mm posterior to the maxillary suture (Fig. 1b). Using a fit-and-fill method, defects were repaired with either 3D-printed scaffolds loaded with 1,000 µM DIPY or bone graft, again ensuring primary stability of reconstruction and avoiding violation of the maxillary sinus membrane.

Alveolar bone graft was created prior to maxillary defect creation by harvesting radial bone from the right rabbit forearm. 10 mm longitudinal incisions were made and soft tissue dissected resulting in exposure of radial bone, for which a 3.5 mm segment was harvested and immersed in saline, using scaffolds as templates. Forearm soft tissue and skin were closed in layered fashion and splint and bandage dressing were applied to rabbit forearm.

Buprenorphine (0.01 mg/kg) and enrofloxacin (5 mg/kg) were administered subcutaneously every 12-hours for up to 48-hours post-op. Animals were given food, *ad libitum*, without any restrictions to their activity. Animals were euthanized after 24 weeks *in vivo* (beyond the completion of craniofacial growth, conservatively estimated at 28–30 weeks)^41,53^ via anesthetic barbiturate overdose. Secondary confirmation of euthanasia was performed via decapitation in accordance with NYU School of Medicine IUCAC standards.

### 3D Scaffold composition and design, dipyridamole concentration

All 3DPBC scaffolds described in this study were composed of 100% β-TCP, designed via computer assisted design (CAD) (RoboCAD 4.3; 3D Inks LLC, Tulsa, OK, USA), and constructed using a custom-built 3D direct-write micro-printer system (Aerotech Inc., Pittsburgh, PA, USA). Colloid gel ink was formulated to achieve solid volume fraction of 46% by combining ceramic powder, ammonium polyacrylate, deionized water, hydroxypropyl methylcellulose, and polyethlenimine^24^. Ink preparation requires ~2 hours. Calvarial scaffolds were printed layer-by-layer in paraffin oil as 10 mm-diameter cylindrical lattices (Fig. 1) with 250 µm struts and 330 µm pore spacing^27,28^ which has previously been determined to result in maximal cranial bone regeneration and effective scaffold degradation kinetics^28^. Alveolar scaffolds were printed in a similar fashion in a 3.5 mm × 3.5 mm × 3.5 mm rectangular lattice^27^. Printing occurs at 7 mm/min resulting in printing of 12 scaffolds in 30 minutes. Scaffolds were then sintered to 1100 °C to densify constructs and eliminate impurities^54^.

Scaffolds were loaded by immersion in 2% bovine collagen solution (Collagen I, Bovine; Corning Inc. Corning, NY, USA) as a carrier and then loaded with 1,000 µM DIPY^28,29,55^. This DIPY concentration has been demonstrated to result in favorable bone regeneration in previous studies^27–29^.

### 3D reconstruction analysis

Rabbit heads were removed *en bloc*, cleared of surrounding soft tissue, and imaged using micropositron emission tomography computed tomography (micro-PET/CT, Siemens Inveon™) scanner at 86 µm slice resolution. CT scans were used to reconstruct animal skulls for evaluation of facial symmetry.

High-resolution micro computed tomography (μCT 40, Scanco medical, Basserdorf, Germany) images were acquired at 18 µm slice resolution for specific areas of interest. These µCT sections were then reconstructed in *Amira* (Version 6.3, Visage Imaging GmbH, Berlin, Germany) to create 3D digital models of the region of interest (ROI) including scaffold or regenerated bone. Bone regeneration, scaffold degradation, and maxillary suture patency were calculated using µCT reconstruction. As previously described^27,28^, 3D reconstructions were uniformly thresholded for bone, scaffold, and soft tissue or empty space using consistent values for bone and scaffold which demonstrate different µCT intensity in Houndsfield units. Then, volume of each component was calculated as a proportion of total volume of the defect (BV/TV or SV/TV). Un-implanted scaffolds also underwent µCT scanning and reconstruction via this method in order to establish a *t* = 0 time point to evaluate scaffold degradation.

µCT images were imported into *ImageJ*^56^ and analyzed for trabecular thickness (Tb.Th) and trabecular spacing (Tb.Sp) using BoneJ Thickness function^39,57,58^. Using ROI manager, region of interest with uniform dimensions of trabecular bone were selected across reconstruction type and thresholded for scaffold, bone, and empty space. Regions of interest were adjusted to nearby anatomical landmarks in order to account for sample orientation and ensure analysis of comparable trabecular regions. µCT analysis was conducted by a single, blinded investigator (MW).

### Histologic sample preparation and analysis

Following whole-face CT imaging, samples were partitioned to isolate region surrounding the calvarial defects. Samples were then dehydrated in a series of alcohol solutions ranging from 70–100% ethanol (EtOH) and embedded in methyl methacrylate resin. The embedded blocks were cut into 250 μm-thick sections using a diamond saw (Isomet 2000, Buehler Ltd., Lake Bluff, IL, USA) with individual slices glued onto acrylic slides and ground on a grinding machine (Metaserv 3000, Buehler, Lake Bluff, IL, USA) under continuous water irrigation with a series of SiC abrasive paper until a thickness of 100 μm were achieved, after which samples were stained in Stevenel’s blue and Van Gieson picro fuchsin to differentiate between bone, scaffold and soft tissue. Sample sections were imaged at low magnification (1 × , 10 µm/pixel) using pathology slide scanner (Aperio Technologies, Vista, CA, USA) and evaluated for suture patency and bone organization including trabecular organization and creation of diploic spaces in the calvarium (Fig. 4a). Samples were qualitatively assessed for presence of ectopic bone formation, scaffold fragmentation, and for any histologic evidence of excess inflammation or morbidity secondary to surgery.

At high magnification (10 × , 1 µm/pixel), samples underwent further histologic characterization to compare organization and vascularization of native, bone graft, and scaffold-regenerated bone including previously-defined features that would indicate maturity and remodeling of bone^40^. In both the alveolus and calvarium, bone regenerated by scaffolds was analyzed by isolating regions between scaffold struts. Bone graft was analyzed by selecting regions on the corresponding side of animals that underwent bone graft reconstruction. Native bone was analyzed by selecting regions from contralateral, un-operated sides of the calvarium and contralateral alveoli.

All histologic counts were performed in *ImageJ*. 3.5 × 2.0 mm sections of bone were assessed for vessel density (vessel/mm^2^). Vessels were identified by morphologic appearance of endothelial cells, clear vessel lumen, or presence of intra-vascular erythrocytes. Average vessel diameter was calculated to assess degree of neo-angiogenesis by measuring the widest dimension of the innermost lumen of each identified vessel (Fig. 5a).

Osteoblast density (osteoblast/mm^2^) was calculated by measuring the number of osteoblasts divided by unit area; osteoblasts were identified by strong cytoplasmic Stevenel’s blue staining, location adjacent to osteoid, and cuboidal cell morphology (Fig. 5b). Osteoclast density (osteoclast/mm^2^). was calculated by measuring the number of osteoclasts per unit area; osteoclasts were identified as multinucleated cells with ruffled border, and location in ‘Howship’s lacunae’ (Fig. 5c). Osteoblast to osteoclast ratio was calculated for each group by dividing average osteoblasts density by average osteoclast density for each condition to assess bone remodeling.

Lastly, to quantify maturity of bone, osteocyte counts were performed. Each animal had n = 4 sagittal histologic sections identified based on consistent presence of premolar incisor root, clear defect region of interest visualization, and maxillary suture visualization. Counts were performed at 10X magnification in 500 × 500 pixel at n = 4–6 counts, where each count region included >90% filling with bone.

### Nanoindentation

Nanoindentation was used to assess mechanical properties of regenerated bone^27^. A second set of slides was processed in a similar manner as for histology slides to ~100 μm, with a sequential 9 to 1 μm diamond suspension (Buehler, Lake Bluff, IL) polishing as necessary for scratch removal. The nanoindentation testing (*n* = 16/specimen) was performed using a nanoindenter (Hysitron TI 950, Minneapolis, MN) equipped with a Berkovich diamond three-sided pyramid probe. The scaffold samples were subject to peak load of 300 µN at a rate of 60 µN/s applied with a holding period of 10 s and an unloading period of 2 s. For each sample, n = 16 indentation points were collected on regenerated bone within scaffold interstices and n = 16 indentations were collected on un-operated bone outside the defect area. Regions of bone were chosen by visualization with light microscope, with scaffold bone selected between regions of scaffold struts and regions of native bone selected distal to the scaffold region and proximal to the maxillary suture in the alveolus, or lateral to the scaffold region in the calvarium. Indentation protocol generated force-displacement curves which was used by indenter software to calculate reduced elastic modulus (*E*_*r*_) and hardness (H) using the following formulae:

1 $${E}_{r}=\frac{\sqrt{\pi }}{2\sqrt{A({h}_{c})}}\times S$$

2 $$H=\frac{{P}_{max}}{A({h}_{c})}$$

where A(*h*_c_) is the contact area at peak load (P_*max*_) and *S* is stiffness^27^.

### Craniofacial morphometric analysis

Morphometric analysis was predicated on the assumption that aberrant craniofacial development secondary to DIPY-3DPBC scaffolds, implanted on the right side of the skull and maxilla, would manifest as asymmetrical growth of the face. Facial development and symmetry at facial maturity was evaluated using 3D dense-surface modeling as previously utilized to characterize patterns of dysmorphic faces in neonatal and pediatric populations^42,59–61^. Single-surface images of rabbit skulls were isolated and prepared from reconstructions of gross CT scans with *Meshlab*^*©*^ and *Blender*^*©*^. Using *Facemark*^*©*^, rabbit faces were then annotated by one individual (MW) with 78 anatomically consistent landmarks as previously reported in studies of rabbit craniofacial morphology^62–64^. A 3D dense-surface model was built using *DSMBuilder*^*©*^, applying the Procrustes algorithm to compute mean landmarks and thin-plate splines so that mean surfaces could be warped to the mean landmarks thus enabling the set of surfaces to be closely aligned. Hence the points on a selected face could be mapped to the closest points on each face enabling the induction of a dense surface correspondence of thousands of points that enabled computation of the mean surface. The differences between corresponding points on each surface and the mean surface were used to obtain the principle components that accounted for 99% of variation and which were then used to build the dense-surface model^43^. For each craniofacial surface, a mirror-image was created and the difference between each sample’s surface and its mirror image calculated an asymmetry index (AI) using previously published methods^65^. Briefly, a reflected form of each surface was generated and left-right landmarks were re-labelled using Facemark^*©*^. *DSMBuilder*^*©*^ was then used to generate a DSM of the original and reflected surface. The asymmetry index was calculated from the Euclidean distances between the DSM of the original and reflected surfaces. Both *Facemark*^*©*^ *and DSMBuilder*^*©*^ were developed by Prof. Hammond and Dr. Suttie^42,59–61^.

Additionally, in an effort to detect localized asymmetry resulting from disruption of cranial or maxillary sutures, two targeted sub-models were created using the above method: an isolated midface model and an isolated calvarial model. The isolated midface model was generated by selectively including landmarks anterior to the intraorbital midline (landmark 5), and the isolated calvarial model was generated by including landmarks posterior to the intraorbital midline (landmark 5). Asymmetry Indices were computed by the method described above.

Facial symmetry analysis was further validated with bilateral cephalometric measurements. Bilateral maxillary (Maxillary Length (ML): anterior corpus ossis incicivi - anterior maxillary premolar; Maxillary Height (MH): anterior corpus ossis incicivi - posterior alveoli I3) and calvarial measurements (Calvarial Length (CL): coronal intercept – occipital intercept; Calvarial Width (CW): bregma - coronal intercept;) were taken for each subject. Each measurement was compared between operated and un-operated sides. Ratio between operated and un-operated sides were compared across group conditions including comparison to un-operated age-matched controls.

### Statistical analysis

Sample size was determined *a priori* using power analysis based on preliminary 8-week experiments. Analysis revealed n=6/group was necessary to achieve power >80% to detect 25% mean differences in bone volume regeneration of scaffolds compared to bone graft with standard deviation of 15%. Alpha (α) was set to 0.05.

3D quantification of bone volume at 24 weeks and histologic osteocyte count analyses were analyzed between scaffold, bone graft, and native bone groups using one-way ANOVA with post-hoc comparison of group means with Tukey’s correction.

Bone volume fraction and scaffold volume fraction analysis between scaffold and bone graft-repaired defects was performed using generalized linear mixed model (GLMM).

Trabecular analysis as well as histologic counts including vessel density, vessel diameter, osteocyte, osteoblast, and osteocyte density were analyzed using one-way ANOVA with post-hoc comparison of groups performed with Tukey’s correction.

For nanoindentation, reduced elastic modulus and hardness of newly formed bone was compared to internal control bone by using a GLMM^27^.

Facial growth was independently assessed through morphometric analysis of asymmetry indices and cephalometrics; both analyses were conducted using one-way ANOVA with post-hoc comparison of groups performed with Tukey’s correction.

µCT volumetric data, histologic quantification, morphometric asymmetry indices, and nanoindentation data are presented as mean value with corresponding 95% CI.

All statistical analyses were performed in SPSS (Version 25.0, IBM Corp., Armonk, NY). All graphs were generated using GraphPad Prism (Version 7.0, GraphPad, La Jolla, CA).

### Statement of significance

In this study, we present what is to our knowledge the longest *in vivo* evaluation of dipyridamole-loaded 3D-printed β –TCP scaffolds in regenerating craniofacial bone in a pediatric rabbit model. Previous investigations of this technology were limited to short-term studies compared to un-filled defects. This is the first study to compare to autogolous bone graft, the standard of care and to directly assess facial symmetry through the age of facial maturity. Using a multi-model evaluation approach, the results of this study favor the osteogenic efficacy, favorable degradation and facial-growth safety of this technology to be translated for craniofacial reconstruction specifically in a pediatric context.

## Supplementary information

Supplmentary Table 1

## Data Availability

The datasets generated during and/or analysed during the current study are available from the corresponding author on reasonable request.
