# Supplementary material for: Dipyridamole-loaded 3D-printed bioceramic scaffolds stimulate pediatric bone regeneration in vivo without disruption of craniofacial growth through facial maturity
Source: Sci Rep. 2019 Dec 5;9:18439. doi: 10.1038/s41598-019-54726-6 (PMC6895073; doi:10.1038/s41598-019-54726-6)
Supplement: Supplementary file 1 — Supplmentary Table 1 [file 41598_2019_54726_MOESM1_ESM.pdf]

“Dipyridamole-loaded 3D-printed bioceramic scaffolds stimulate pediatric bone regeneration in vivo without disruption of craniofacial growth through facial maturity” Supplemental Items

Maxime M. Wang BA[1, 2*], Roberto L. Flores MD[2], Lukasz Witek MSci PhD[1], Andrea Torroni MD[2], Amel Ibrahim MD PhD[1,2], Zhong Wang BS[1], Hannah A. Liss BA[1], Bruce N. Cronstein MD[3], Christopher D. Lopez MD[1], Samantha G. Maliha MD MSci[2], Paulo G. Coelho DDS PhD[1,2,4]

[1] Department of Biomaterials & Biomimetics, NYU College of Dentistry, 433 1st Avenue, New York NY 10010

[2] Hansjörg Wyss Department of Plastic Surgery, NYU Langone Health, 307 E 33rd St, New York NY 10016

[3] Department of Medicine, NYU Langone Health, 550 1st Avenue, New York NY 10016

[4] Department of Mechanical Engineering, NYU Tandon School of Engineering, 6 MetroTech Center, Brooklyn NY 11201

Corresponding Author

Mr. Maxime M. Wang, BA
307 E 33rd St
New York, NY 10016
+1 (212) 263-3030
maxime.wang@nyulangone.org

Group	N
Scaffold-repaired Animals (2 Unilateral Defects/ Animal)	8
Calvarial Defects (n=8)	
Alveolar Defects (n=8)	
Bone-graft-repaired Animals (2 Unilateral Defects/ Animal)	8
Calvarial Defects (n=8)	
Alveolar Defects (n=8)	
Un-operated age-matched control Animals	6

Supplemental Table 1: Breakdown of experimental groups including number of animals and defects per group
